# Effectiveness of acupuncture on urinary retention after radical hysterectomy for cervical cancer in China: a systematic review and meta-analysis

**DOI:** 10.3389/fmed.2024.1375963

**Published:** 2024-06-06

**Authors:** Yong Guo, Haixia Pan, Siyang Chen, Minne Tian, Yanmei Huang, Ying Zhou

**Affiliations:** ^1^The First Clinical College of Guangzhou University of Chinese Medicine, Guangzhou, Guangdong, China; ^2^Huizhou First Maternal and Child Health Care Hospital, Guangzhou, Guangdong, China; ^3^Shenzhen Hospital of Integrated Traditional Chinese and Western Medicine, Guangzhou, Guangdong, China; ^4^The First Affiliated Hospital of Guangzhou University of Chinese Medicine, Guangzhou, Guangdong, China

**Keywords:** acupuncture, electroacupuncture, cervical cancer, radical hysterectomy, urinary retention, systematic review, meta-analysis

## Abstract

**Background:**

Cervical cancer is one of the most common malignant tumors worldwide. Radical hysterectomy is the first choice for patients with early-stage cervical cancer. Studies have suggested that acupuncture may be a more effective therapy for the prevention and treatment of urinary retention after radical hysterectomy.

**Objective:**

To systematically evaluate the clinical efficacy of acupuncture in the prevention and treatment of urinary retention after radical hysterectomy.

**Methods:**

We searched the Cochrane library, Web of science, PubMed, Embase, Chinese Biomedical Literature Database, Wanfang database, Wipu database, China National Knowledge Infrastructure Database and ClinicalTrials.gov with the time from inception until December 2023, to collect randomized controlled studies on the clinical efficacy of acupuncture for prevention and treatment of urinary retention after radical hysterectomy. Literature meeting criteria was screened for data extraction. Quality evaluation was performed according to the Cochrane Handbook for Systematic Reviews of Interventions. And meta-analysis was performed using RevMan5.3 and stata14.0 software.

**Results:**

22 Randomized controlled trials with 1,563 patients, 854 in treatment group and 709 in control group, were included totally. Meta-analysis results showed that: the total effective rate in acupuncture group was higher than that in control group, with a statistically significant difference [relative risk (RR)] = 1.43, 95% confidence interval (CI 1.22, 1.68), *p* < 0.0001; the rate of urinary tract infection in acupuncture group was lower than that in control group, with a statistically significant difference [RR] = 0.23, 95% CI (0.07, 0.78), *p* < 0.05; the time of indwelling urinary catheter was reduced in acupuncture group compared with control group, with a statistically significant mean difference = −3.45, 95% CI (−4.30, −2.59), *p* < 0.00001; the incidence of urinary retention was lower in acupuncture group than in control group, and the difference was statistically significant [RR = 0.37, 95% CI (0.27, 0.50), *p* < 0.00001]; the residual urine volume was reduced in acupuncture group compared with control group, with a statistically significant mean difference = −50.73, 95% CI (−63.61, −7.85), *p* < 0.00001.

**Conclusion:**

Acupuncture treatment based on conventional therapy can better prevent and improve urinary retention after radical hysterectomy for cervical cancer, could be a better option for them.

**Systematic review registration:**

Registered by PROSPERO and the registration number is CRD42023452387.

## 1 Introduction

Cervical cancer threatens the health and lives of women worldwide and has the second highest incidence of female malignant tumors (1). In recent years, with the development of economic and medical care, the incidence of cervical cancer in developed countries has declined significantly, but the incidence and mortality of cervical cancer still remain high in developing countries (2). Currently, the incidence of cervical cancer in China accounts for 19% of the global incidence, and about 72.7% of the patients are early stage IA1-IIA2 patients. For these early-stage patients, the International Society of Gynecology and Obstetrics recommends radical hysterectomy (RH) as a first therapy option. However, this therapy could damage the pelvic autonomic nerves, and complications such as abnormalities in bladder function, colorectal peristalsis, and sexual function often occur. One of the most common postoperative complications is urinary retention(UR), which occurs in 8 to 80% of cases (3). UR greatly affects the therapeutic effect of surgery and postoperative quality of life of patients, and also adds considerable difficulty to clinical diagnosis and treatment. Therefore, the prevention and treatment of postoperative UR is of great clinical significance.

At present, to address the problem of postoperative UR, the main therapy ways in modern medicine include aseptic care, indwelling urinary catheter, intermittent catheterization, and bladder function training (4). Clinical studies have found that compared with modern medicine, acupuncture in traditional medicine is more effective in preventing and treating postoperative UR, and acupuncture treatment is more favored by doctors and patients because of its safety and convenience (5–7). However, the persuasiveness needs to be further improved due to factors such as small sample size and variable study quality in each study. The aim of this study is to systematically evaluate the clinical efficacy of acupuncture in the prevention and treatment of postoperative UR for cervical cancer and to provide an evidence-based basis for clinical treatment.

## 2 Materials and methods

### 2.1 Research registration

We compliant Preferred Reporting Items for Systematic Review and Meta-Analysis Protocols (PRISMA-P) guidelines to conduct this study (8). Our protocol of study has been registered in the International Prospective Systematic Registration Review (PROSPERO) on August 17, 2023 and the registration number is CRD42020139497.

### 2.2 Search strategy

Computer searched a total of nine databases, including the Cochrane library, Web of science, PubMed, Embase, Chinese Biomedical Literature Database, Wanfang database, Wipu database, China National Knowledge Infrastructure Database and ClinicalTrials.gov with the search time from their inception until December 2023. The language of this systematic review is limited to Chinese and English only. A combination of subject terms and free words will be used in the search. (See Supplementary Data for details of the specific search terms and algorithms used).

### 2.3 Study selection

All retrieved literature was imported into EndNote (V.X9.0) Reference Manager. Literature screening was performed independently by 2 researchers according to the inclusion and exclusion criteria. After removing duplicates, an initial screening was performed based on the title, abstract and keywords of the literature to exclude studies that did not meet the criteria, and those that might meet the criteria were reviewed in full text to determine the inclusion of the literature. In case of disagreement, a third person was consulted and a joint decision was made.

#### 2.3.1 Inclusion criteria

1. Study type: Published randomized controlled trials (RCTs);
2. Study participants: Patients who were definitively diagnosed with cervical cancer and underwent RH, or patients who were definitively diagnosed with UR after RH for cervical cancer;
3. Diagnostic criteria: There are recognized diagnosis or inclusion and exclusion criteria in the original literature;
4. Interventions: The control group used conventional therapeutic care, including aseptic care, indwelling urinary catheterization, intermittent catheterization, and bladder function training, etc.; the treatment group was based on the control group, plus acupuncture treatment, including ordinary acupuncture and electroacupuncture.
5. Outcome indicators: The main indicators were the total effective rate of treatment and the urinary tract infection rate. The secondary indicators were the residual urine volume, and time of indwelling urinary catheter and the incidence of UR.

#### 2.3.2 Exclusion criteria

1. Non-clinical efficacy studies such as reviews, case reports, and animal studies;
2. Studies in which the treatment group intervention did not focus on acupuncture treatment or in which the control group intervention included acupuncture;
3. Studies for which the full text could not be accessed or for which the data were incomplete;
4. Repeatedly published studies.

### 2.4 Data extraction

After pre-extracting, data extraction was performed independently by 2 researchers with the optimal data extraction form. Followed by cross-checking, and a third person was consulted in case of disagreement and then a joint decision was made. Data extraction included study title, first author, publication date, study source, diagnostic criteria, inclusion exclusion criteria, sample size, age, study methodology, intervention, duration of treatment, acupuncture points taken, and outcome indicators.

### 2.5 Quality assessment of included studies

Risk of bias was assessed using the methodology recommended by the Cochrane Handbook for Systematic Reviews of Interventions (9). It consists of seven main items: (1) randomized sequence generation (selective bias); (2) allocation concealment (selective bias); (3) blinding of participants, caregivers, or those delivering the intervention (implementation bias); (4) blinding of outcome assessors (measurement bias); (5) missing outcome data (attritional bias); (6) selection of reported outcomes (reporting bias), and (7) other sources of bias (other bias). Each entry was assessed as “low risk,” “unclear risk” or “high risk.” In case of disagreement, seek the opinion of a third person and make a joint decision.

### 2.6 Statistical analysis

Meta-analysis of the extracted data was performed using RevMan 5.3 and Stata 14.0 software. The relative risk (RR) and its 95% confidence interval (CI) were used as effect indicators for dichotomous variable data. Mean difference (MD) and its 95% CI were used as effect indicators for the measurement data, and *p* ≤ 0.05 indicated that the difference was statistically significant.

*p*-value and I2 were used to quantify the magnitude of heterogeneity. When *p* > 0.1 and I2 ≤ 50%, indicating no statistical heterogeneity among studies, a fixed-effects model was used; when *p* ≤ 0.1 and I2 > 50%, indicating statistical heterogeneity among studies, a random-effects model was used instead, and the source of the heterogeneity was analyzed.

The stability of the study results was judged by sensitivity analysis. If the results of Meta-analysis included 10 or more studies, the harbored method could be used to detect publication bias for dichotomous variables, and Egger’s method was used to detect publication bias for continuous variables, and *p* < 0.05 would be considered statistically significant.

## 3 Results

### 3.1 Study selection

**Figure 1:**
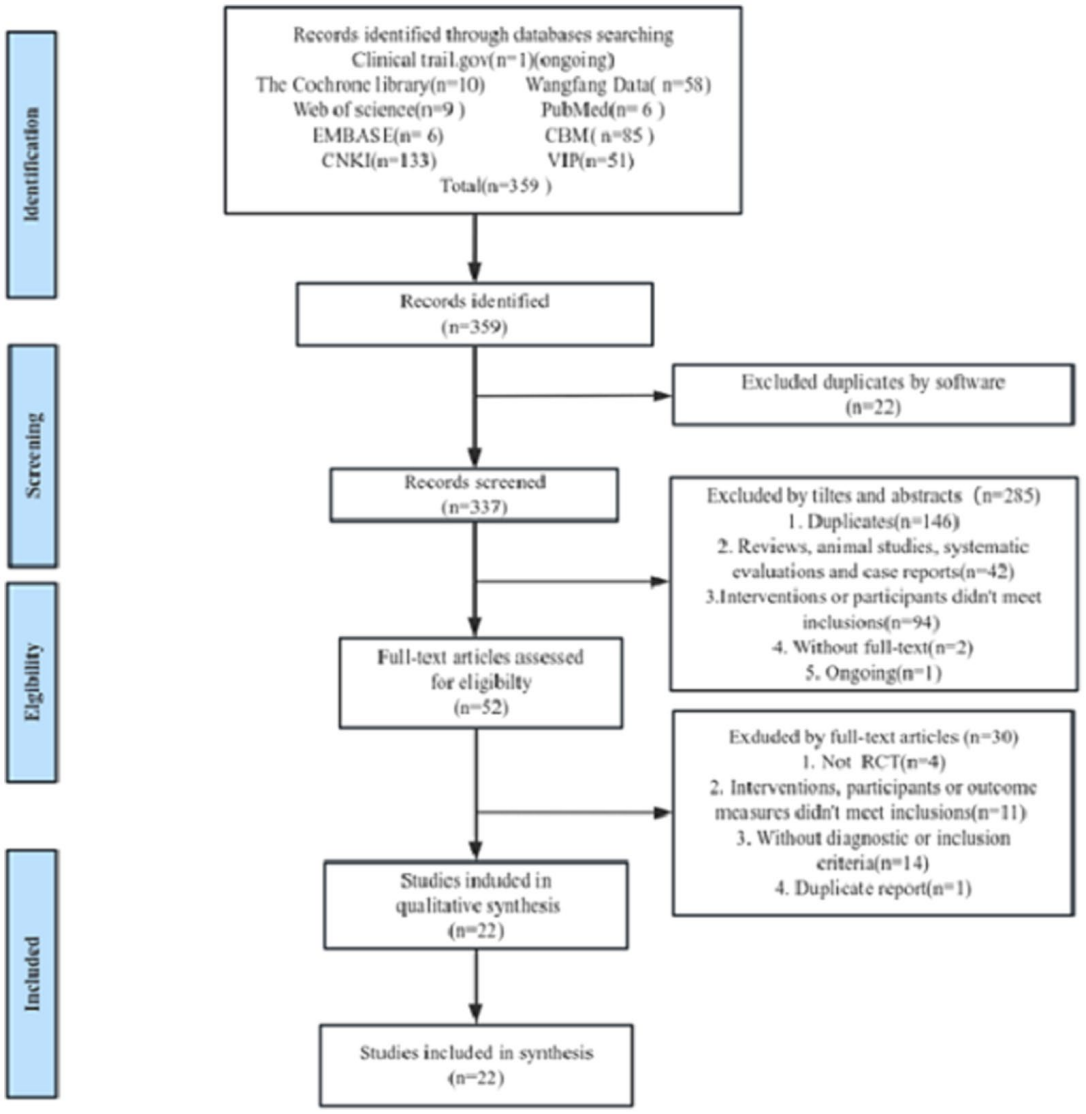
The flow diagram of study selection.

359 literatures were retrieved initially. 168 duplicate literatures were excluded. After initial screening based on the title, abstract and keywords of the literature, studies that did not meet the criteria were removed, and 52 potentially eligible literatures were reviewed in full text. Eventually, a total of 22 studies were included after excluding 30 studies that were not randomized controlled trials, did not have clear inclusion and exclusion criteria, had no eligible outcome indicator, did not meet the criteria for the participants or interventions, and were reported repeatedly. The study selection flow diagram is shown in Figure 1.

### 3.2 Characteristics of the included studies

The characteristics of the included studies are shown in Table 1. We included a total of 22 studies (10–31) all of which were RCTs, totally 1,563 participants (854 in the treatment group and 709 in the control group). Although we did not limit the countries, all 22 studies were from China. In terms of participants, 10 studies (16, 19, 21, 22, 24–28, 31) included patients with prophylactic treatment UR after radical hysterectomy for cervical cancer and 12 studies (10–15, 17, 18, 20, 23, 29, 30) included patients with confirmed postoperative UR for cervical cancer; in terms of trial groups, Eight studies (11, 12, 14, 15, 19, 23, 25) had three groups, only two groups meeting the criteria were extracted from six studies (11, 12, 14, 19, 23, 25) only one study (15) met the criteria for all three groups, and the other (16) included only two groups due to missing data on the control group; two studies (27, 31) had four groups, one study (27) included 2 acupuncture groups and 1 control group, and another (31) included all four groups; 1 study had 5 groups and included 4 groups that met the criteria; and the remaining 11 studies all had only 2 groups. For acupuncture treatment, 11 studies (10–12, 14–17, 25, 26, 30, 31) used acupuncture and another 11 (13, 18, 21–23, 27–29) used electroacupuncture. In terms of adjunctive therapeutic modalities shared by the treatment and control groups in the studies, one study (23) used herbal medicine; two studies (12, 31) used biofeedback training; three studies (11, 20, 25) used low-frequency or mid-frequency electrical stimulation; two studies (16, 18) used injectable medications; and the rest were conventional treatments. The characteristics of the included studies are shown in Tables 1, 2.

**Table 1:** The characterization of the 22 included studies.

Studies	Randomization method	Sample size	Age	Intervention		Treatment time	Outcomes
		T\C	T\C	Treatment group	Control group		
Sun LP et al., 2023 (10)	NA	56\56	42.09 ± 6.18\42.35 ± 6.35	A + TCM + GT	TCM + GT	14 days	①②③④
Tang BZ et al., 2020 (11)	Random number table	20\19	51.05 ± 9.53\52.37 ± 7.59	A + LFES+GT	LFES+GT	10 days	①③
Luo YL et al., 2019 (12)	Random number table	33\33	44.75 ± 7.21	A + BBT + GT	BBT + GT	21 days	①③④
Zhao SJ et al., 2015 (13)	NA	30\30	63.7 ± 3.2\64.2 ± 2.7	EA + GT	GT	20 days	①③
Shen et al., 2015 (14)	NA	30\30	46.13 ± 8.78\47.23 ± 7.83	A + Neostigmine+GT	Neostigmine+GT	12 days	①③
Hu H et al., 2012 (15)	Random number table	20\19\19⑥	45.55 ± 5.84\43.63 ± 8.10	A + GT	GT	7 days	①③
			\41.95 ± 5.38				
Zi D et al., 2011 (16)	NA	30\30	38.27 ± 8.1/39.23 ± 5.16	A + Terazosin+GT	Terazosin+GT	3 days	③⑤
Zhou X et al., 2010 (17)	NA	35\33	38–57	EA + GT	GT	5 days	①
Zhou YM et al., 2003 (18)	Coin-flip method	40\34	34.53\33.41	EA + GT	GT	5 days	①
Chen GY et al., 2022 (19)	Random number table	49\49	52.16 ± 4.09\52.74 ± 4.37	EA + GT	GT	36 days	③⑤
Cao Y et al., 2022 (20)	NA	34\33	49.16 ± 8.25\48.59 ± 8.76	EA + LFES+GT	LFES+GT	12 days	③④⑤
Guo XW et al., 2020 (21)	NA	15\16	43.27 ± 12.00\48.94 ± 8.22	EA + GT	GT	10 days	①③
Yang DR et al., 2019 (22)	NA	36\36	45.11 ± 13.68\46.10 ± 12.88	EA + GT	GT	5 days	②③④⑤
Wang H et al., 2019 (23)	Random number table	11\11	48.91 ± 6.35\45.73 ± 9.08	EA + GT	GT	7 days	①③④
Peng Y et al., 2019 (24)	NA	32\32	49.0 ± 19.9\48.9 ± 19.7	EA + GT	GT	30 days	①②③④
Yang ZJ et al., 2017 (25)	Random number table	56\56	46.1 ± 9.7\45.7 ± 11.3	A + GT	GT	12 days	①⑤
Ding XH et al., 2017 (26)	Area group randomization	30\30\30⑦	49.70 ± 9.24\47.43 ± 10.13	SBA\A + GT	GT	10 days	③④
			\48.70 ± 9.27				
Yang ZJ et al., 2016 (27)	Random number table	30\30\30⑧	46.36 ± 9.66\46.13 ± 9.72	EA + GT	GT	10 days	⑤
			\45.65 ± 11.25				
Ye PZ et al., 2014 (28)	Computer randomization	74\33	46.92 ± 13.69\46.26 ± 12.89	EA + GT	GT	10 days	①③④
Pan XH et al., 2012 (29)	NA	30\30	46 ± 19	EA + TDP + GT	TDP + GT	5 days	①
Liu HM et al., 2012 (30)	NA	18\14	45 ± 16	A + GT	GT	5 days	③⑤
Liu HY et al., 2009 (31)	NA	31\25	46	A + GT	GT	12 days	③⑤
		35\30⑨		A + BBT + GT	BBT + GT		

*NA, only randomization is mentioned in the literature and no specific randomization method is described; T, treatment group; C, control group; A, Acupuncture; EA, Electroacupuncture; TCM, traditional Chinese medicine; GT, general treatment, including intermittent catheterization, indwelling urinary catheter and bladder function training, etc.; LFES, low-frequency electric stimulation; BBT, biofeedback training; SBA, the scalp and body acupuncture; TDP, stands for “Teding Dianci Pu,” a specific type of electromagnetic wave. ①The total effective rate of treatment; ②the rate of urinary tract infection; ③the residual urine volume; ④the time of indwelling urinary catheter; ⑤the incidence of UR; ⑥The study included the control group (19), treatment 1 group (19), and treated 2 group(20); ⑦The study included the control group, the acupuncture group, and the cephalic-body-acupuncture group; ⑧The study included the control group with the two acupuncture groups (with different acupuncture points); ⑨The study included the control group (25, acupuncture group (31), biofeedback training group (30), and acupuncture + biofeedback training group (35), respectively, and compared the control group with the acupuncture group, and the acupuncture group with the acupuncture + biofeedback training group, respectively different).

**Table 2:** The characterization of interventions of the 22 included studies.

Studies	Intervention		Treatment schedule
	Treatment group	Control group	
Sun LP et al., 2023 (10)	Acupuncture at CV3, ST28, SP6, CV4, BL67 and others.	TCM + general treatment	Postoperative treatment was started for 30 min once a day for 14 days. Both groups were given YiqiHuoxueLishui decoction.
Tang BZ et al., 2020 (11)	Acupuncture at the Li, Tui, and Kan positions around the belly button and others.	general treatment	Postoperative treatment was started for 25 min twice a day for 10 days.
Luo YL et al., 2019 (12)	Acupuncture at GV20, CV3, CV4, CV6.	general treatment	Treatment was started on the 4th postoperative day for 30 min once a day for 21 days.
Zhao SJ et al., 2015 (13)	Electroacupuncture at CV3, CV4, ST28, ST36, SP9, SP6, LR3, LI4.	general treatment	Postoperative treatment was started for 30 min once a day for 20 days.
Shen et al., 2015 (14)	Acupuncture at GV20, KI3, LU7, CV3, CV6, CV4, SP6, SP9, ST36.	general treatment	Postoperative treatment was started for 30 min once a day for 12 days.
Hu H et al., 2012 (15)	Acupuncture at BL31, BL32, BL33, BL34, SP6, SP9, BL54.	general treatment	Group 1 started treatment on the 8th day after surgery; group 2 started on the 15th day; both groups treatment was once a day for 30 min for 7 days.
Zi D et al., 2011 (16)	Acupuncture at CV3, CV6, CV4.	Oral Terazosin hydrochloride 2 mg.	Treatment was started on the 7th postoperative day for 30 min once a day for 3 days.
Zhou X et al., 2010 (17)	Electroacupuncture at BL31, BL32, BL33, BL34, SP10, SP9, SP6, ST36, BL28.	general treatment	Postoperative needling was started for 20 min twice a day for 5 days.
Zhou YM et al., 2003 (18)	Electroacupuncture at SP6, SP10, CV3.	Intramuscular injection of neostigmine 1 mL.	Postoperative treatment was started for 30 min once a day for 5 days.
Chen GY et al., 2022 (19)	Electroacupuncture at Iliohypogastric nerve, iliohypogastric inguinal nerve, pubic nerve,0.5 inch paracentesis of the tip of the coccyx.	Pelvic floor muscle training.	Treatment was started 1 week after surgery and continued for 36 days at 1 h 3 times a week.
Cao Y et al., 2022 (20)	Electroacupuncture at BL31, BL32, BL33, BL34,BL23, BL35, SP6.	low-frequency bladder+general treatment	Treatment was started 1 week after surgery and continued for 12 days at 40 min 3 times a week.
Guo XW et al., 2020 (21)	Electroacupuncture at SP6, SP9, ST36, CV6, CV4,ST28, CV3.	general treatment	Treatment was started on the 1st postoperative day for 15 min once a day for 10 days.
Yang DR et al., 2019 (22)	Electroacupuncture at CV2, CV4, BL28, CV3, ST36, SP6.	general treatment	Treatment was started on the 5th postoperative day for 40 min once a day for 5 days.
Wang H et al., 2019 (23)	Electroacupuncture at SP6, SP9, CV4, CV6, ST36, ST28, CV3.	general treatment	Postoperative treatment was started for 30 min once a day for 7 days.
Peng Y et al., 2019 (24)	Electroacupuncture at BL28, SP6, CV4, ST36, CV3.	general treatment	Treatment was started on the 5th postoperative day for 40 min once a day for 30 days.
Yang ZJ et al., 2017 (25)	Acupuncture at CV12,ST25,ST28,LR3, SP6,ST36,SP9.	general treatment	Treatment was started on the 3rd postoperative day for 20 min once a day for 12 days.
Ding XH et al., 2017 (26)	Acupuncture at BL28, BL32, SP6, SP9; other group added the foot sensory area of the head based on the former.	general treatment	Postoperative needling was started for 30 min once a day for 10 days.
Yang ZJ et al., 2016 (27)	Electroacupuncture at SP9; the other group at SP6.	general treatment	Treatment was started on the 4th postoperative day for 20 min once a day for 10 days.
Ye PZ et al., 2014 (28)	Electroacupuncture at SP6, SP9, CV4, CV6, ST36, CV3.	general treatment	Treatment was started on the 4th postoperative day for 30 min once a day for 10 days.
Pan XH et al., 2012 (29)	Electroacupuncture at SP6, ST36, CV4, BL32, BL28.	TDP + general treatment	Postoperative needling was started for 30 min once a day for 5 days.
Liu HM et al., 2012 (30)	Acupuncture at the foot sensory and genital areas of the head, BL22, BL24, BL25, BL26, BL60.	general treatment	Postoperative needling was started for 30 min once a day for 10 days.
Liu HY et al., 2009 (31)	Acupuncture at BL31, BL32, BL33, BL34.	Bioelectrical stimulation+general treatment	Treatment was started on the 3rd postoperative day for 30 min once a day for 12 days.

### 3.3 Quality assessment of studies

**Figure 2:**
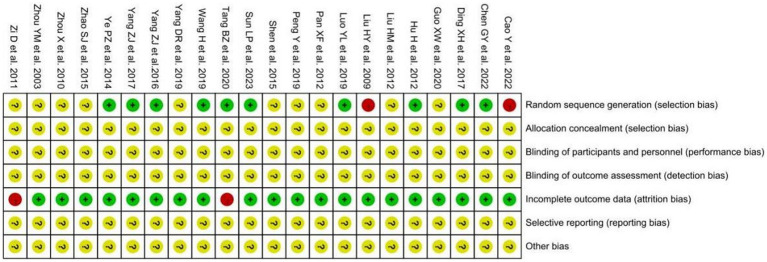
The summary chart of risk of bias for included studies.

**Figure 3:**
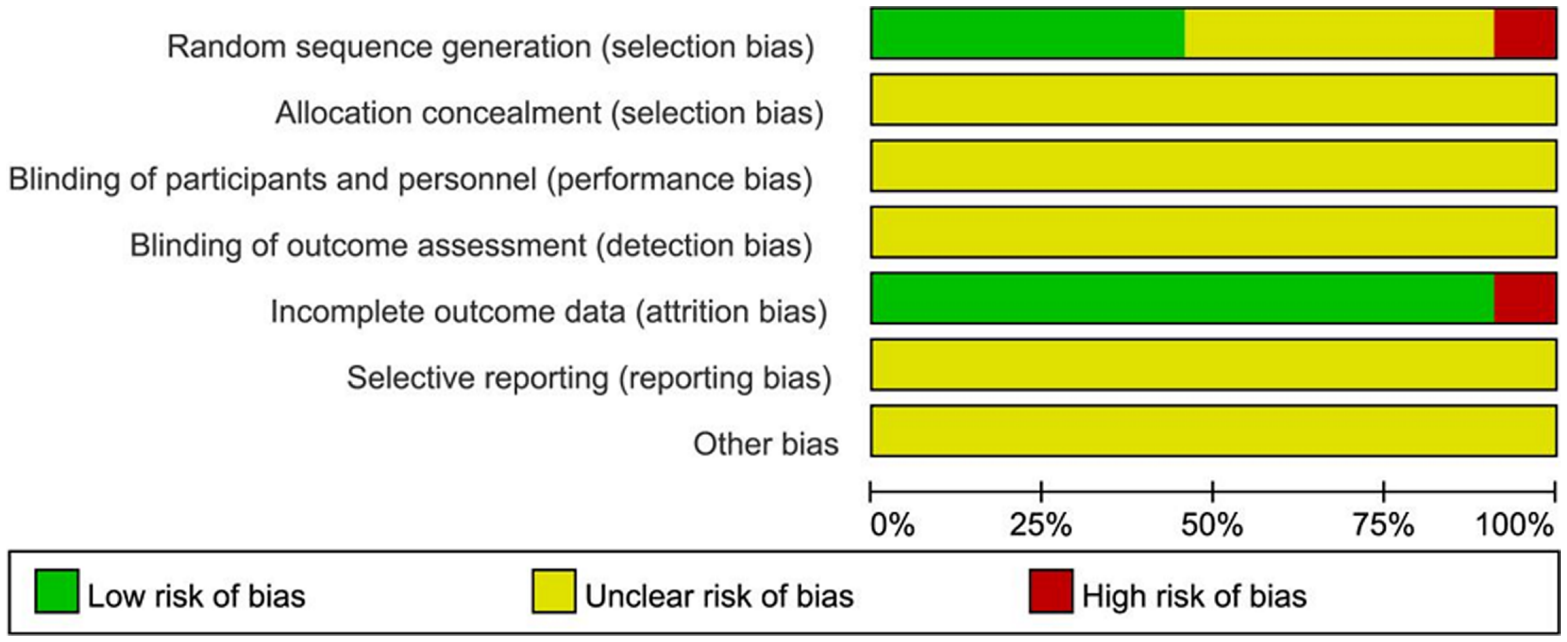
The percentage risk of bias graph for included studies.

Risk of bias was assessed for the included studies using the methods recommended by the Cochrane Handbook for Systematic Reviews of Interventions. (1) Random allocation methods: the included studies all mentioned random allocation, among which seven studies (11, 12, 15, 19, 23, 25, 27) used random number table method, one (10) randomized grouping using SPSS23.0 software, one (26) district group randomization, and one (28) used computer randomization, so they were evaluated as low risk; two mentioned grouping according to the order of medical consultation or operation order with (20, 31) machine grouping, so they were evaluated as high risk; the remaining 10 items (13, 14, 16–18, 21, 22, 24, 29, 30) did not specify the method of randomization, so the mean was unclear. (2) Allocation concealment and blinding of implementers and participants, only 1 study (31) mentioned single-blind study methodology, but because it did not specifically describe how it was operationalized; therefore, the implementation of allocation concealment and blinding in 22 studies was evaluated as unclear. (3) Completeness of results, only 1 study (16) did not mention the replacement tube data of the control group, so it was evaluated as high risk; the rest of the studies did not have incomplete results data, so they were evaluated as low risk. (4) Selective outcome reporting and other biases, 22 were unclear about their selective reporting of outcomes and other biases, so they were all evaluated as unclear. The risk of bias assessment is shown in Figures 2, 3.

### 3.4 Meta-analysis results

#### 3.4.1 The total effective rate of treatment

**Figure 4:**
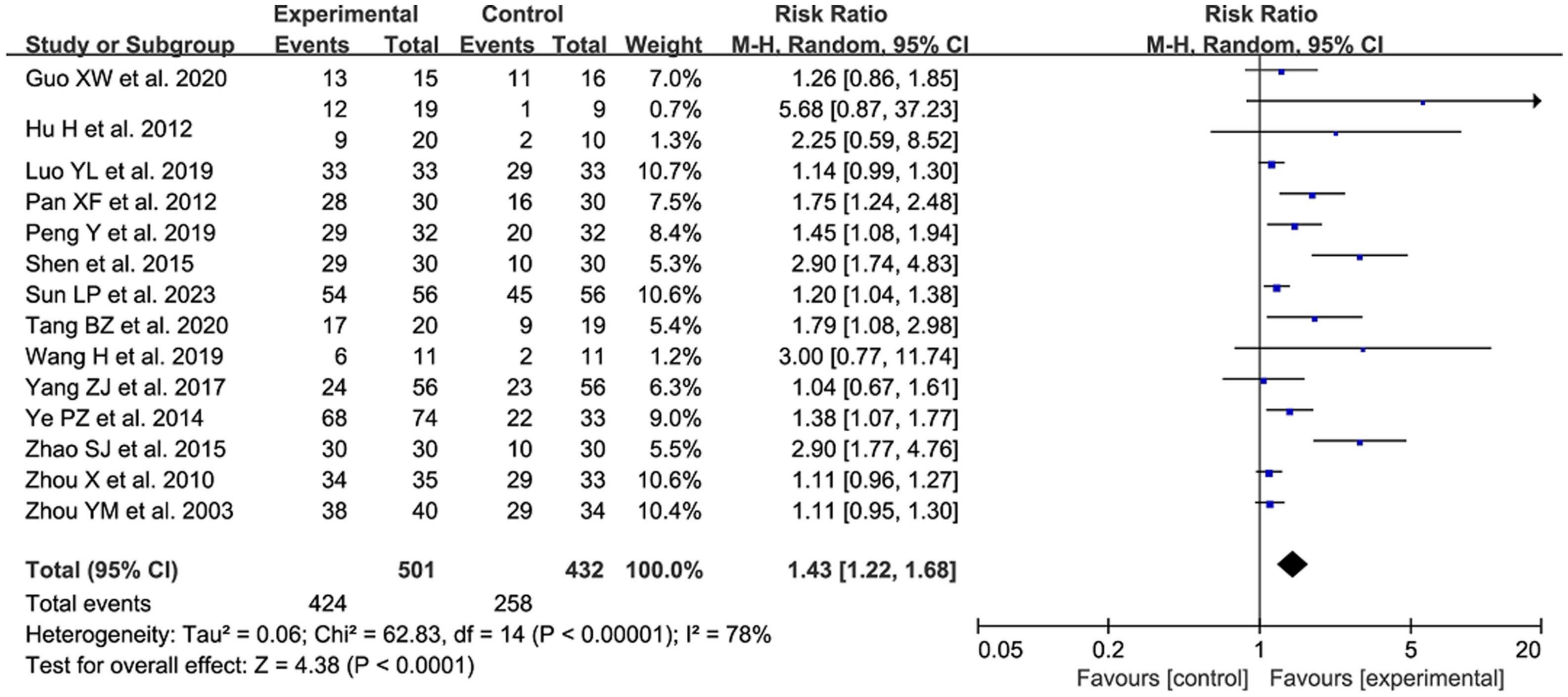
The forest plot of the total effective rate. Relative Risk values <1 shows measures of in favor of control group (left side) and Relative Risk values >1 are in favor of treatment group (right side).

A total of 14 studies (10–16, 22–24, 26, 28, 30, 31) containing 15 sets of data reported the total effective rate of acupuncture for the clinical treatment of postoperative UR. After combining and analyzing the heterogeneity among the studies, it was found that the heterogeneity was large (*p* < 0.00001, I^2^ = 78%), so the random effect model was used for the analysis. The results showed that compared with the control group, the acupuncture group could better improve the clinical symptoms of postoperative UR in patients with cervical cancer, which was statistically significant [RR = 1.43, 95% CI (1.22, 1.68), *p* < 0.0001; Figure 4].

#### 3.4.2 The rate of urinary tract infections

**Figure 5:**
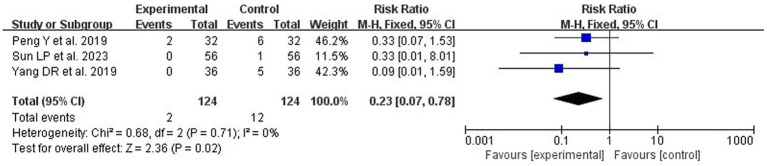
The forest plot of urinary tract infection rates. Relative Risk values <1 shows measures of in favor of control group (left side) and Relative Risk values >1 are in favor of treatment group (right side).

A total of 3 studies (10, 22, 24) reported post-treatment urinary tract infections. After combining and analyzing the heterogeneity among the studies, no heterogeneity was found (*p* = 0.71, I^2^ = 0%), so the fixed-effect model was used for analysis. The results showed that acupuncture treatment reduced the risk of postoperative urinary tract infection in patients compared with the control group, which was statistically significant [RR = 0.23, 95% CI (0.07, 0.78), *p* = 0.02; Figure 5].

#### 3.4.3 The residual urine volume

**Figure 6:**
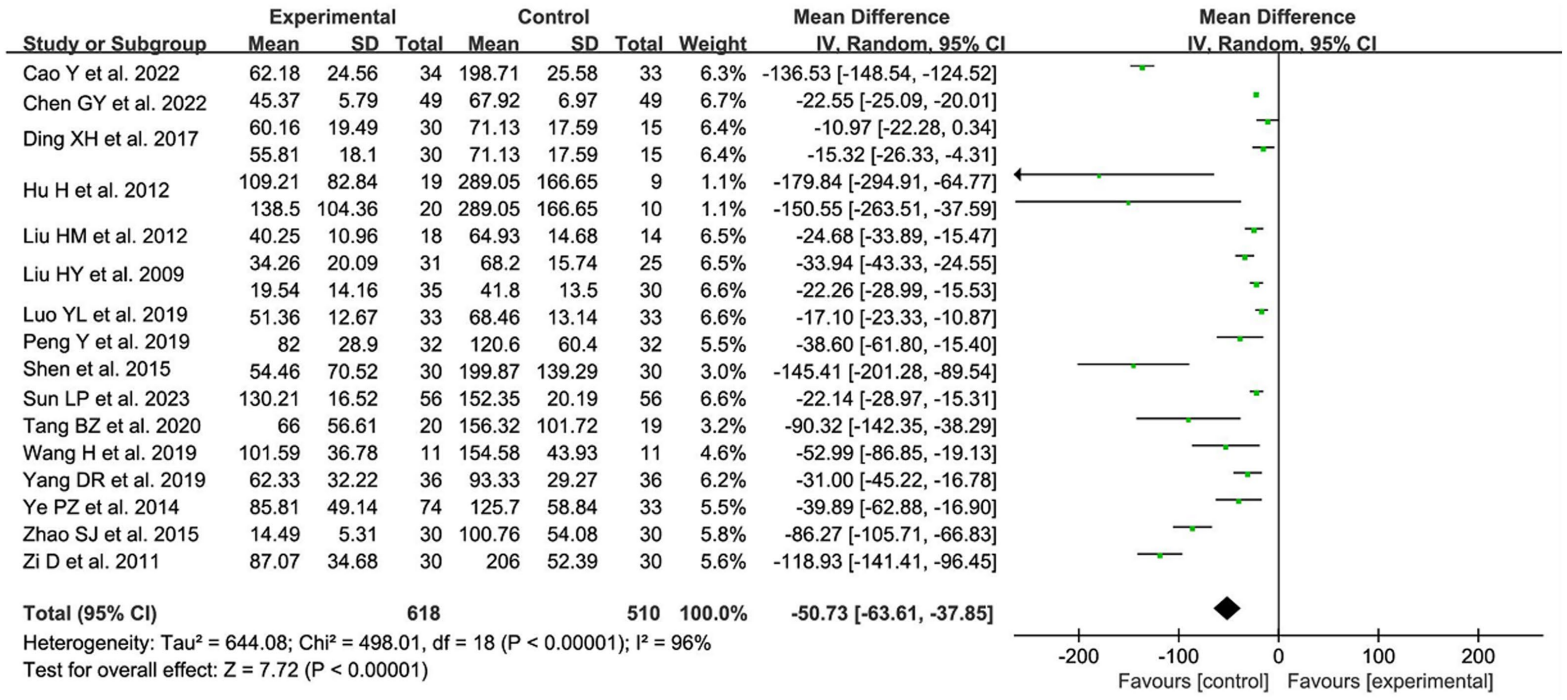
The forest plot of residual urine volume. Mean Difference values <0 shows measures of in favor of control group (left side) and Mean Difference values >0 are in favor of treatment group (right side).

A total of 16 studies (10–16, 19, 20, 22–24, 26, 28, 30, 31) with 19 data sets reported on residual urine volume after treatment. After combining and analyzing the heterogeneity among the studies, the heterogeneity was great large (*p* < 0.00001, I^2^ = 96%), so a random effects model was used for the analysis. The results showed a statistically significant reduction in residual urine volume in patients after acupuncture treatment compared with the control group [MD = −50.73, 95% CI (−63.61, −37.85), *p* < 0.00001; Figure 6].

#### 3.4.4 The time of indwelling urinary catheter

**Figure 7:**
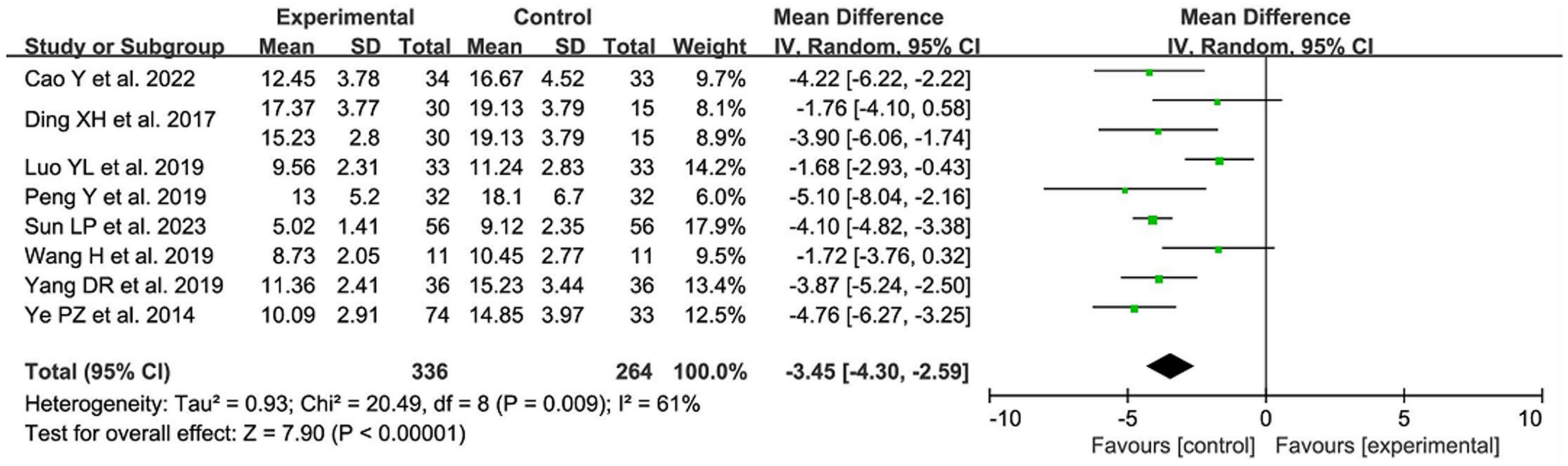
The forest plot of the time of indwelling urinary catheter. Mean Difference values <0 shows measures of in favor of control group (left side) and Mean Difference values >0 are in favor of treatment group (right side).

A total of 8 studies (10, 12, 20, 22–24, 26, 28) with 9 data sets reported the time of indwelling urinary catheter after acupuncture for postoperative UR. After combining and analyzing the heterogeneity among the studies, heterogeneity existed (*p* = 0.009, I^2^ = 61%), so random effects model was used for the analysis. The results showed a statistically significant reduction in the patients’ time of indwelling urinary catheter after acupuncture treatment compared with the control group [MD = −3.45, 95% CI (−4.30, −2.59), *p* < 0.00001; Figure 7].

#### 3.4.5 The incidence of UR

**Figure 8:**
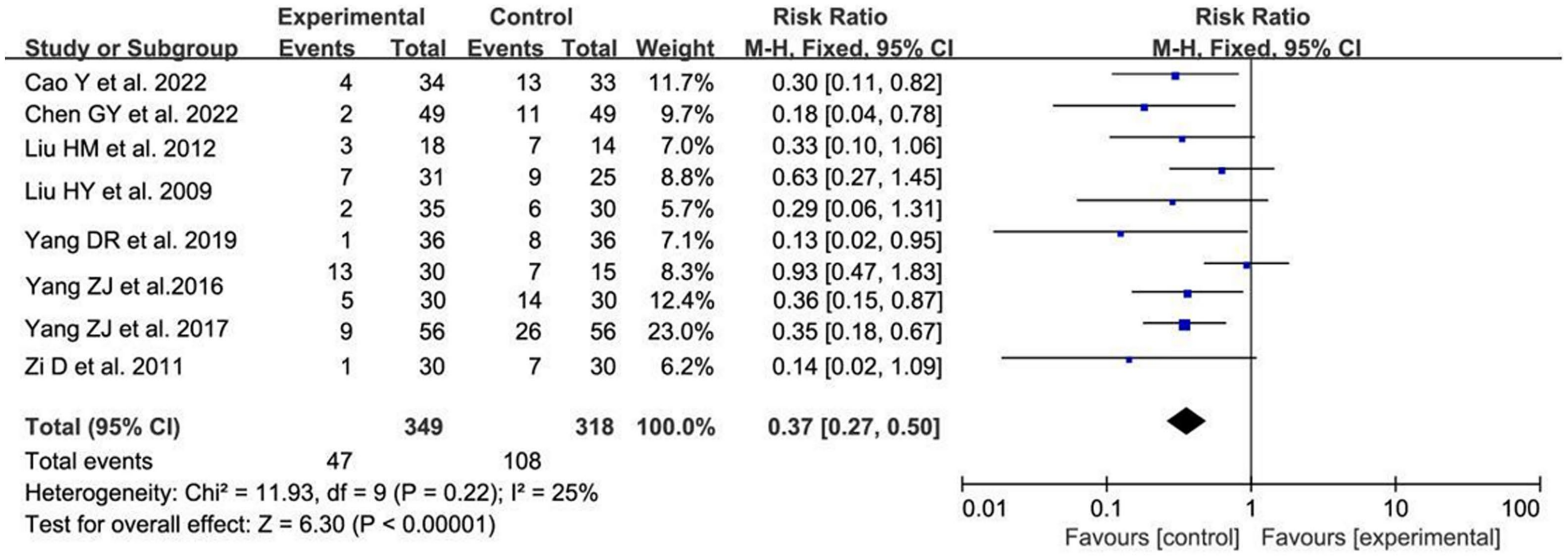
The forest plot of the incidence of UR. Relative Risk values <1 shows measures of in favor of control group (left side) and Relative Risk values >1 are in favor of treatment group (right side).

A total of 8 studies (16, 19, 20, 22, 25, 27, 30, 31), 10 sets of data reported the incidence of UR after acupuncture treatment. After combining and analyzing the heterogeneity between studies, the heterogeneity was small (*p* = 0.22, I^2^ = 24%), so the fixed effect model was used to analyze. The results showed that acupuncture treatment reduced the incidence of UR in postoperative patients compared with the control group, which was statistically significant [RR = 0.37, 95% CI (0.27, 0.50), *p* < 0.00001; Figure 8].

#### 3.4.6 Sensitivity analysis

The analysis of changing the scale of the effect, the cut-and-complement method and item-by-item exclusion study revealed that the total effectiveness rate, the rate of urinary tract infections, the rate of urinary tract infection, residual urine volume, the time of indwelling urinary catheter, and the rate of UR of the acupuncture group were steadily and statistically improved when compared with that of the control group (see Supplementary Data for detailed data).

#### 3.4.7 Subgroup analysis

##### 3.4.7.1 Acupuncture group and electroacupuncture group

Subgroup analyses were conducted for the four outcome indicators of total effective rate, residual urine volume, time of indwelling urinary catheter and incidence of UR according to the subgroups of treatment using acupuncture or electroacupuncture. None of the subgroup analyses were able to reduce inter-study heterogeneity, so they were analyzed using a random-effects model. The results showed that both the acupuncture and electroacupuncture groups significantly increased the total effective rate, reduced the residual urine volume and shortened the time of indwelling urinary catheter, and none of them differed between groups (*p* > 0.1; see Supplementary Data for details of the specific data phase.)

##### 3.4.7.2 Confirmed postoperative UR group and postoperative prophylactic treatment of UR group

Subgroups were divided into two groups according to whether postoperative UR was diagnosed or not after radical hysterectomy for cervical cancer, and subgroup analyses were performed on the four outcome indexes of total effective rate, residual urine volume, time of indwelling urinary catheter and incidence of UR. Also, none of the subgroup analyses were able to reduce inter-study heterogeneity, so they were analyzed using a random-effects model. The results showed that both groups were effective in improving the symptoms of postoperative UR, and there was no between-group difference in any indicators (*P* > 0.1; see Supplementary Data for details of the specific data phase).

#### 3.4.8 Reporting bias

**Figure 9:**
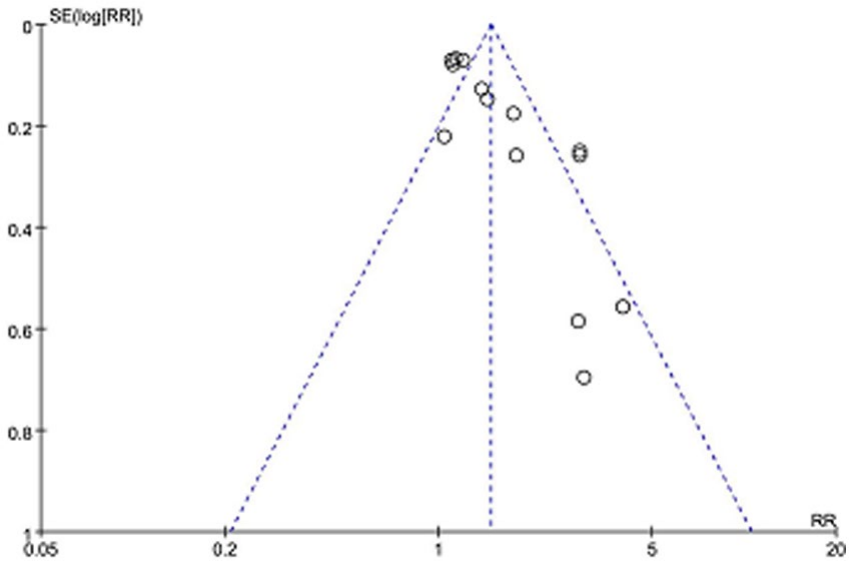
The funnel plot of total clinical effectiveness.

The funnel plot of reporting bias was drawn using the total effective rate as an indicator (Figure 9). stata14.0 software was further applied to quantitatively analyze its reporting bias using the harbored method. The results showed that there was no reporting bias in the literature, which was statistically significant (*t* = 1.01, *p* > 0.1).

## 4 Discussion

In our study, 22 randomized controlled trials were combined. Meta-analysis showed that the total clinical effectiveness rate was significantly higher in the treatment group compared with the control group [RR = 1.43, 95% CI (1.22, 1.68), *p* < 0.0001]; acupuncture treatment also significantly reduced the rate of urinary tract infections in patients [RR = 0.23, 95% CI (0.07, 0.78), *p* = 0.02]. When subgroups were analyzed, it was found that both acupuncture and electroacupunctur groups showed significant improvement in the treatment of UR after radical hysterectomy for cervical cancer compared with the control group, and there was no between-group difference (*p* > 0.1). Analysis of the acupuncture point-taking characteristics of the included study revealed that the selected points were mainly based on the bladder channel of foot-taiyang, the spleen channel of foot-shaoyin, the yin channel and the liver channel of foot-jueyin, etc., among which the points of Sanyinjiao, Yinlingquan, Guanyuan, Qihai, Zhusanli, Pangguangshu, Zhongji, and Ciliao were the most commonly used main points.

Postoperative UR in cervical cancer is generally defined as those who cannot urinate on their own or can urinate on their own but the residual urine volume is greater than 100 mL for 14 days or more after surgery (32). Currently, RH is considered to be the mainstay of treatment for early-stage cervical cancer (33). The scope of surgical resection includes the uterus, fallopian tubes, upper vagina, main ligament and complete clearance of paravaginal and pelvic lymph nodes, and during the resection process, the pelvic autonomic nerves will be destroyed, and some of the nerves that innervate the bladder will be severed, which, together with the perioperative psychosomatic condition, the change of the anatomical position of the pelvic organs, surgical anesthesia and postoperative pain, lead to bladder contraction and sensory dysfunction, as well as voiding disorders and UR (34–39). Studies have shown that UR caused by mechanical injury to the urethral muscle due to excessive intraoperative stretching, inhibition of the micturition reflex by anesthetic drugs, and postoperative wound pain or postural changes in the position of the body that are afraid of or unaccustomed to urination can be recovered by itself in less than 60 h, so that if the amount of residual urine still does not return to normal in 1 week after the operation, it suggests that there is a presence of damage to the peripheral nerves that innervate the bladder (40).

Traditional acupuncture mainly acts on meridians and acupoints. With the wide application of acupuncture in a variety of diseases, people have become more and more curious about the mechanism of action of acupuncture in treating diseases, and a number of related studies have emerged. Some scholars have suggested that the neuroanatomical and neurophysiological basis of acupoints is a prerequisite for the acupuncture effect (41). Clinical studies have found that acupuncture at Sanyinjiao can be effective in improving UR (42, 43). The anatomical basis of Sanyinjiao acupoint, which is characterized by the presence of the saphenous and tibial nerves in the fascial layer of the skin, which are connected to the sacral medulla oblongata, and the tibial nerves, whose course coincides with that of the bladder meridian in the lower extremities, provides a theoretical basis for the effective treatment of UR and other voiding disorders at the Sanyinjiao (44). For patients with postoperative UR, Yang Wenting (32) analyzed the results of urodynamics and residual urine volume in the bladder after acupuncture at the Baliao point, and found that acupuncture could both reduce the hyperreflexia of the urethra muscle during the storage phase and elevate the pressure of the urethra muscle during the voiding phase, indicating that acupuncture has a bi-directional regulating effect, and proposed that acupuncture may work through the mechanism of repairing sympathetic or parasympathetic nerve injury and eliminating bladder edema. In addition, compared with the participants with UR who had the urge to urinate, the effective rate of acupuncture treatment was significantly lower in the subjects without the urge to urinate, indicating that the more severe the nerve damage, the worse the efficacy of acupuncture, and also laterally verifying the mechanism that acupuncture can treat postoperative UR after cervical cancer by repairing the damaged nerves (32). It has also been found in animal model studies that electroacupuncture at Guanyuan point may improve UR after spinal cord injury by promoting the repair of spinal cord neural pathways, improving the ultrastructure of detrusor cells and tissues, inhibiting apoptosis, increasing detrusor excitability and contractility, and reestablishing the excitatory-contractile voiding function of the bladder (45).

A number of other studies have found that acupuncture improves UR through humoral regulation. Cheng et al. (46) showed that electroacupuncture the points of Sanyinjiao, Yinlingquan and Weizhong could treat acute urinary retention (AUR), and its mechanism of action was related to the elevation of intravesical pressure and the content of adenosine triphosphate (ATP) in bladder tissues. Zhang et al. (47) suggested that acupuncture protected against AUR-induced bladder dysfunction by decreasing urinary ATP concentration and bladder pressure in AUR humans and animals through a reduction in ATP release from transient receptor potential vanilloid 1(TRPV1) channels. Recent studies (48) have also shown that electroacupunture can improve the urinary dysfunction in rats with neurogenic UR, which may be related to its effect in inhibiting the activation of 3-phosphoinositide-dependent protein kinase 1 /protein kinase B(PDK1/Akt) pathway, promoting hyperpolarization activated cyclic nucleotide-gated cation channel 4(HCN4) mediated detrusor excitatory contraction and urinary electrical signal activation.

All in all, on the one hand, acupuncture may regulate bladder function by repairing the nerves damaged during surgery and enhancing the role of signals transmitted to the nervous system; on the other hand, it may regulate the body fluids, thus producing endogenous substances that can improve UR (49, 50).

Recently, the number of Meta-analyses on acupuncture for postoperative UR after cervical cancer is increasing. As in the recently published meta-analysis (51), we have more stricter selection criteria in comparison, which can lead to a higher quality of selected literature; in addition, we included two types of studies using acupuncture or electroacupuncture treatment, which allows us to compare the therapeutic efficacy of the two methods for postoperative UR at the same time; and lastly, we analyzed the preventive and therapeutic roles of acupuncture in the occurrence of postoperative UR after radical hysterectomy for cervical cancer, and we found that acupuncture treatments are effective in preventing and treating postoperative UR.

Although the blinding of the study was unmentioned in the majority of included studies, the stability of the analyzed results was good and no publication bias was detected. Besides, there is no denying that this study still has some limitations. First, most of the included studies did not specify the method of randomization, which may have affected the quality of the study; second, significant between-study heterogeneity was detected in the analysis. When we performed subgroup analyses and to reduce heterogeneity, heterogeneity between subgroups was also significant; therefore, we were unable to find the source of heterogeneity based on quantitative analyses.

## 5 Conclusion

Acupuncture treatment can not only significantly improve the clinical symptoms of postoperative UR in cervical cancer, but also be associated with the prevention of its occurrence. Therefore, it can be a better treatment modality for the prevention and treatment of UR after radical hysterectomy for cervical cancer. However, few blinded clinical trials have demonstrated the efficacy of acupuncture in the prevention and treatment of postoperative UR after radical hysterectomy for cervical cancer, which points to the direction for future research.

## Data availability statement

The original contributions presented in the study are included in the article/Supplementary material, further inquiries can be directed to the corresponding author.

## Author contributions

YG: Writing – review & editing, Writing – original draft, Visualization, Data curation. HP: Writing – review & editing. SC: Writing – review & editing, Methodology, Data curation. MT: Writing – review & editing, Software, Methodology, Formal analysis, Data curation. YH: Writing – review & editing, Methodology, Investigation. YZ: Writing – review & editing.
