# Supplementary material for: Effectiveness of acupuncture on urinary retention after radical hysterectomy for cervical cancer in China: a systematic review and meta-analysis
Source: Front Med (Lausanne). 2024 Jun 6;11:1375963. doi: 10.3389/fmed.2024.1375963 (PMC11187101; doi:10.3389/fmed.2024.1375963)
Supplement: Supplementary file 1 [file Data_Sheet_1.docx]

1. **Search strategy**

**1.1 Chinese Biomedical Literature Database(CBM)**

1.1.1 MeSH search

| 主题词 | 款目词 |
| --- | --- |
| 宫颈肿瘤 | 子宫颈癌、宫颈癌、子宫颈肿瘤、宫颈部肿瘤、宫颈癌根治术 |
| 随机对照试验、随机 | 随机对照临床试验、随机临床试验； |
| 尿潴留 | / |
| 针灸、针刺、电针 | / |

1.1.2 Advanced search

#1 "宫颈肿瘤"[常用字段:智能] OR "子宫颈癌"[常用字段:智能] OR "宫颈癌"[常用字段:智能] OR "宫颈部肿瘤"[常用字段:智能] OR "子宫颈肿瘤"[常用字段:智能] OR "宫颈癌根治术"[常用字段:智能]

#2 "尿潴留"[常用字段:智能]

#3 "针灸"[常用字段:智能] OR "针刺"[常用字段:智能] OR "电针"[常用字段:智能]

#4 "随机对照试验"[常用字段:智能] OR "随机对照临床试验"[常用字段:智能] OR "随机临床试验"[常用字段:智能] OR "随机"[常用字段:智能]

#5 #1 AND #2 AND #3 AND #4

((((((("尿潴留"[常用字段:智能]) AND (("宫颈肿瘤"[常用字段:智能] OR "子宫颈癌"[常用字段:智能] OR "宫颈癌"[常用字段:智能] OR "宫颈部肿瘤"[常用字段:智能] OR "子宫颈肿瘤"[常用字段:智能] OR "宫颈癌根治术"[常用字段:智能])))) AND (("针灸"[常用字段:智能] OR "针刺"[常用字段:智能] OR "电针"[常用字段:智能]))))) AND (("随机对照试验"[常用字段:智能] OR "随机对照临床试验"[常用字段:智能] OR "随机临床试验"[常用字段:智能] OR "随机"[常用字段:智能])))

**1.2 China National Knowledge Infrastructure Databaseand(CNKI)**

（主题：宫颈肿瘤）OR（主题：子宫颈癌）OR（主题：宫颈癌）OR（主题：宫颈部肿瘤）OR（主题：子宫颈肿瘤）OR（主题：宫颈癌根治术）AND（主题：尿潴留）AND（主题：针灸）OR（主题：针刺）OR（主题：电针）

**1.3 Wanfang database**

#1 主题:(宫颈肿瘤) or 主题:(子宫颈肿瘤) or 主题:(子宫颈癌) or 主题:(宫颈癌) or 主题:(宫颈部肿瘤) or 主题:(宫颈癌根治术)

#2 主题:(尿潴留)

#3 主题:(针刺) or 主题:(电针) or 主题:(针灸)

#4 主题:(随机对照试验) or 主题:(随机临床试验) or 主题:(随机对照临床试验) or 主题:(随机)

#5 #1 and #2 and #3 and #4

**1.4 Wipu database**

(M=(宫颈肿瘤 or 子宫颈肿瘤 or 子宫颈癌 or 宫颈癌 or 宫颈部肿瘤 or 宫颈癌根治术)) and (M=尿潴留) and (M=(针刺 or 电针 or 针灸)) and (U=(随机对照试验 or 随机临床试验 or 随机对照临床试验 or 随机))

**1.5 PubMed**

1.5.1 MeSH search

| MeSH | Entry Terms |
| --- | --- |
| Uterine Cervical Neoplasms | Cervical Neoplasm, Uterine  Neoplasm, Uterine Cervical  Uterine Cervical Neoplasm  Neoplasms, Cervical  Cervical Neoplasms  Cervical Neoplasm  Neoplasms, Cervix  Cervix Neoplasm  Neoplasm, Cervix  Cervix Neoplasms  Cancer of the Uterine Cervix  Cancer of the Cervix  Cervical Cancer  Cancer, Cervical  Cervical Cancers  Uterine Cervical Cancer  Cancer, Uterine Cervical  Cervical Cancer, Uterine  Uterine Cervical Cancers  Cancer of Cervix  Cervix Cancer  Cancer, Cervix |
| Urinary Retention | Retention, Urinary |
| acupuncture  acupuncture and moxibustion  electroacupuncture | / |

1.5.2 Advanced search

(((Uterine Cervical Neoplasms) or (Cervical Neoplasm, Uterine) or (Neoplasm, Uterine Cervical) or (Uterine Cervical Neoplasm) or (Neoplasms, Cervical) or (Cervical Neoplasms) or (Cervical Neoplasm) or (Neoplasms, Cervix) or (Cervix Neoplasm) or (Neoplasm, Cervix) or (Cervix Neoplasms) or (Cancer of the Uterine Cervix) or (Cancer of the Cervix) or (Cervical Cancer) or (Cancer, Cervical) or (Cervical Cancers) or (Uterine Cervical Cancer) or (Cancer, Uterine Cervical) or (Cervical Cancer, Uterine) or (Uterine Cervical Cancers) or (Cancer of Cervix) or (Cervix Cancer) or (Cancer, Cervix)) AND ((Urinary Retention) or (Retention, Urinary))) AND ((acupuncture) or (acupuncture and moxibustion) or (electroacupuncture))

**1.6 Embase**

1.6.1 MeSH search

| MeSH | Entry Terms |
| --- | --- |
| ’uterine cervix cancer’ | Ca cervix  cancer of the cervix  cancer of the cervix uteri  cancer of the uterine cervix  cancer, uterine cervix  carcinogenesis of the cervix  cervical cancer  cervical cancerogenesis  cervical carcinogenesis  cervical malignancies  cervical malignancy  cervix ca  cervix cancer  cervix cancer, recurrent  cervix cancer, uterine  cervix cancerogenesis  cervix carcinogenesis  cervix malignancies  cervix malignancy  cervix malignancy, recurrent  cervix uteri cancer  cervix uterus cancer  malignancies of the cervix  malignancy of the cervix  neoplasma cervicis recurrens  neoplasma cervicis uteri recurrens  recurrent cancer of the cervix  recurrent cervix cancer  recurrent cervix malignancy  uterine cervical cancer  uterine cervix cancer, recurrent  uterine cervix malignancy, recurrent  uterine neck cancer  uterus cervix cancer  uterine cervix cancer |
| urine retention | ischuria  retention, urine  urinary retention  urine retention |
| acupuncture  electroacupuncture  acupuncture and moxibustion | acupuncture therapy  shonishin  acupuncture  acupuncture, electric  electric acupuncture  electrical acupoint stimulation  electrical acupuncture  electro-acupuncture  electrode acupuncture  electronic acupuncture |

1.6.2 Advance search

('ca cervix'/exp OR 'ca cervix' OR 'cancer of the cervix'/exp OR 'cancer of the cervix' OR 'cancer of the cervix uteri'/exp OR 'cancer of the cervix uteri' OR 'cancer of the uterine cervix'/exp OR 'cancer of the uterine cervix' OR 'cancer, uterine cervix'/exp OR 'cancer, uterine cervix' OR 'carcinogenesis of the cervix'/exp OR 'carcinogenesis of the cervix' OR 'cervical cancer'/exp OR 'cervical cancer' OR 'cervical cancerogenesis'/exp OR 'cervical cancerogenesis' OR 'cervical carcinogenesis'/exp OR 'cervical carcinogenesis' OR 'cervical malignancies'/exp OR 'cervical malignancies' OR 'cervical malignancy'/exp OR 'cervical malignancy' OR 'cervix ca'/exp OR 'cervix ca' OR 'cervix cancer'/exp OR 'cervix cancer' OR 'cervix cancer, recurrent'/exp OR 'cervix cancer, recurrent' OR 'cervix cancer, uterine'/exp OR 'cervix cancer, uterine' OR 'cervix cancerogenesis'/exp OR 'cervix cancerogenesis' OR 'cervix carcinogenesis'/exp OR 'cervix carcinogenesis' OR 'cervix malignancies'/exp OR 'cervix malignancies' OR 'cervix malignancy'/exp OR 'cervix malignancy' OR 'cervix malignancy, recurrent'/exp OR 'cervix malignancy, recurrent' OR 'cervix uteri cancer'/exp OR 'cervix uteri cancer' OR 'cervix uterus cancer'/exp OR 'cervix uterus cancer' OR 'malignancies of the cervix'/exp OR 'malignancies of the cervix' OR 'malignancy of the cervix'/exp OR 'malignancy of the cervix' OR 'neoplasma cervicis recurrens'/exp OR 'neoplasma cervicis recurrens' OR 'neoplasma cervicis uteri recurrens'/exp OR 'neoplasma cervicis uteri recurrens' OR 'recurrent cancer of the cervix'/exp OR 'recurrent cancer of the cervix' OR 'recurrent cervix cancer'/exp OR 'recurrent cervix cancer' OR 'recurrent cervix malignancy'/exp OR 'recurrent cervix malignancy' OR 'uterine cervical cancer'/exp OR 'uterine cervical cancer' OR 'uterine cervix cancer, recurrent'/exp OR 'uterine cervix cancer, recurrent' OR 'uterine cervix malignancy, recurrent'/exp OR 'uterine cervix malignancy, recurrent' OR 'uterine neck cancer'/exp OR 'uterine neck cancer' OR 'uterus cervix cancer'/exp OR 'uterus cervix cancer' OR 'uterine cervix cancer'/exp OR 'uterine cervix cancer') AND ('ischuri' OR 'retention, urine'/exp OR 'retention, urine' OR 'urinary retention'/exp OR 'urinary retention' OR 'urine retention'/exp OR 'urine retention') AND ('acupuncture'/exp OR acupuncture OR 'electroacupuncture'/exp OR electroacupuncture OR 'acupuncture and moxibustion' OR 'acupuncture therapy'/exp OR 'acupuncture therapy' OR 'shonishin'/exp OR shonishin OR 'acupuncture, electric'/exp OR 'acupuncture, electric' OR 'electric acupuncture'/exp OR 'electric acupuncture' OR 'electrical acupoint stimulation'/exp OR 'electrical acupoint stimulation' OR 'electrical acupuncture'/exp OR 'electrical acupuncture' OR 'electro acupuncture'/exp OR 'electro acupuncture' OR 'electrode acupuncture'/exp OR 'electrode acupuncture' OR 'electronic acupuncture'/exp OR 'electronic acupuncture')

**1.7 The Cochrane library**

1.7.1 MeSH search

| MeSH | Entry Terms |
| --- | --- |
| Uterine Cervical Neoplasms | Neoplasm, Uterine Cervical; Cervical Neoplasm, Uterine; Uterine Cervical Neoplasm; Cervix Neoplasms; Neoplasms, Cervical; Cervix Neoplasm; Neoplasm, Cervix; Neoplasms, Cervix; Cervical Neoplasms; Cervical Neoplasm; Cervical Cancers; Cancer of Cervix; Uterine Cervical Cancer; Cervix Cancer; Cancer, Cervix; Cancer, Uterine Cervical; Uterine Cervical Cancers; Cancer of the Uterine Cervix; Cervical Cancer; Cervical Cancer, Uterine; Cancer, Cervical; Cancer of the Cervix |
| Urinary Retention | Retention, Urinary |
| acupuncture  acupuncture and moxibustion  Electroacupuncture | / |

1.7.2 Advance search

(‘Uterine Cervical Neoplasms’ OR ‘Neoplasm, Uterine Cervical’ OR ‘Cervical Neoplasm, Uterine’ OR ‘Uterine Cervical Neoplasm' OR 'Cervix Neoplasms' OR 'Neoplasms, Cervical' OR 'Cervix Neoplasm' OR 'Neoplasm, Cervix' OR 'Neoplasms, Cervix' OR 'Cervical Neoplasms' OR 'Cervical Neoplasm' OR 'Cervical Cancers' OR 'Cancer of Cervix' OR 'Uterine Cervical Cancer' OR 'Cervix Cancer' OR 'Cancer, Cervix' OR 'Cancer, Uterine Cervical' OR 'Uterine Cervical Cancers' OR 'Cancer of the Uterine Cervix' OR 'Cervical Cancer' OR 'Cervical Cancer, Uterine' OR 'Cancer, Cervical' OR 'Cancer of the Cervix’) AND (‘Urinary Retention’ OR ‘Retention, Urinary’) AND (acupuncture OR ‘acupuncture and moxibustion’ OR electroacupuncture)

**1.8 Web of science**

1.8.1 Advanced search

(((Uterine Cervical Neoplasms) or (Cervical Neoplasm, Uterine) or (Neoplasm, Uterine Cervical) or (Uterine Cervical Neoplasm) or (Neoplasms, Cervical) or (Cervical Neoplasms) or (Cervical Neoplasm) or (Neoplasms, Cervix) or (Cervix Neoplasm) or (Neoplasm, Cervix) or (Cervix Neoplasms) or (Cancer of the Uterine Cervix) or (Cancer of the Cervix) or (Cervical Cancer) or (Cancer, Cervical) or (Cervical Cancers) or (Uterine Cervical Cancer) or (Cancer, Uterine Cervical) or (Cervical Cancer, Uterine) or (Uterine Cervical Cancers) or (Cancer of Cervix) or (Cervix Cancer) or (Cancer, Cervix)) AND ((Urinary Retention) or (Retention, Urinary))) AND ((acupuncture) or (acupuncture and moxibustion) or (electroacupuncture))

**1.9 ClinicalTrials.gov**

Urinary Retention, Acupuncture Therapy | Cervix Cancer

1. **Sensitivity analysis**
   1. **Changing the scale of the effect**

| Indicators | RR 95%CI | OR 95%CI |
| --- | --- | --- |
| The total effective rate | 1.43[1.22,1.68](P＜0.0001) | 5.93[3.21,11.00](P＜0.00001) |
| the rate of urinary tract infections | 0.23[0.07,0.78](P=0.02) | 0.20[0.06,0.74](P=0.02) |
| The incidence of UR | 0.37[0.27,0.50](P＜0.00001) | 0.26[0.17,0.39](P＜0.00001) |
| Indicators | MD | SMD |
| The residual urine volume | -61.85[-81.04,-42.65](P＜0.00001) | -1.70[-2.13，-1.27]（P＜0.00001） |
| The time of indwelling urinary catheter | -3.45[-4.30，-2.59](P＜0.00001) | -1.10[-1.45,-0.75](P＜0.00001) |


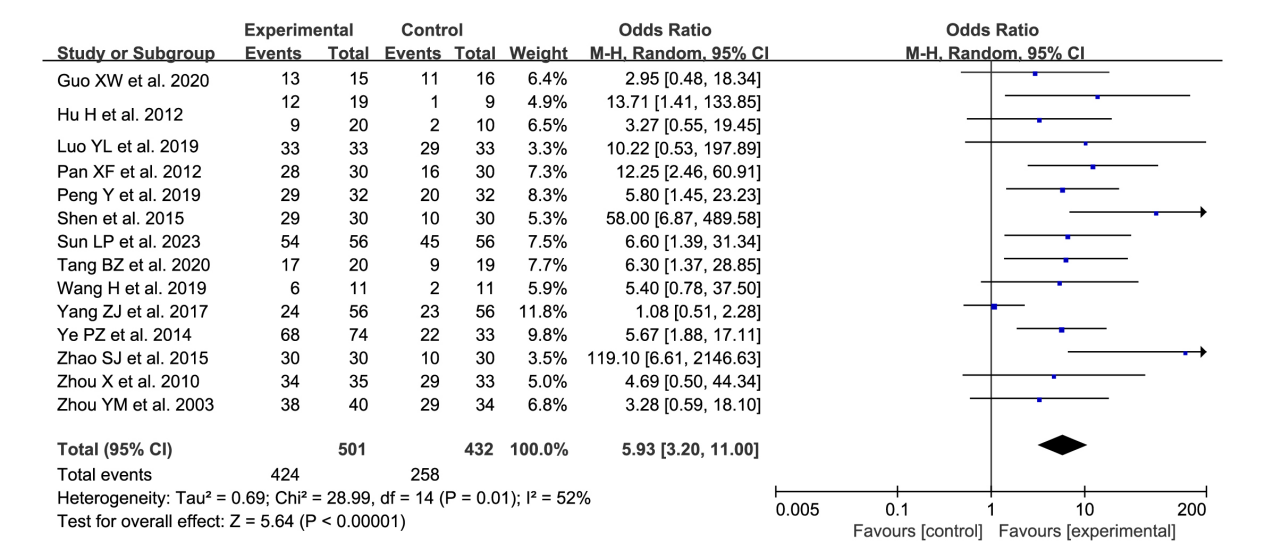


Fig 2.1.1. The forest plot of the total effective rate(OR).

**
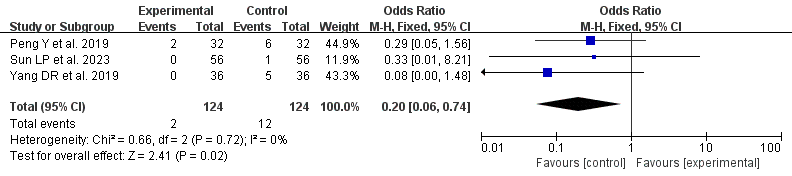
**

Fig 2.1.2. The forest plot of urinary tract infection rates(OR).


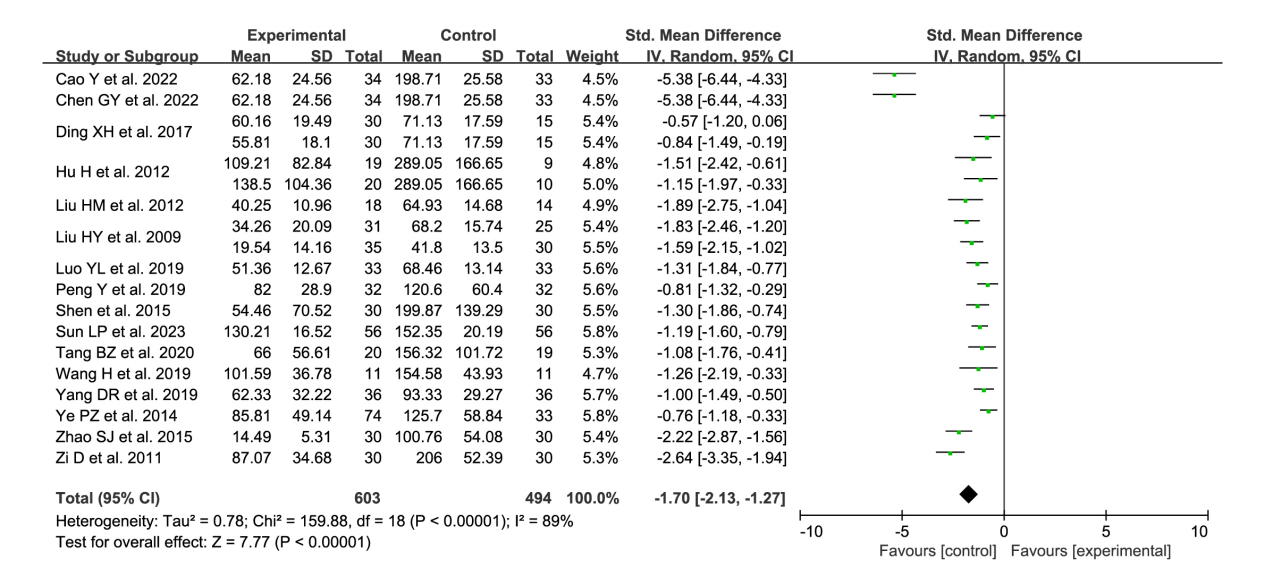


Fig 2.1.3. The forest plot of residual urine volume(SMD).


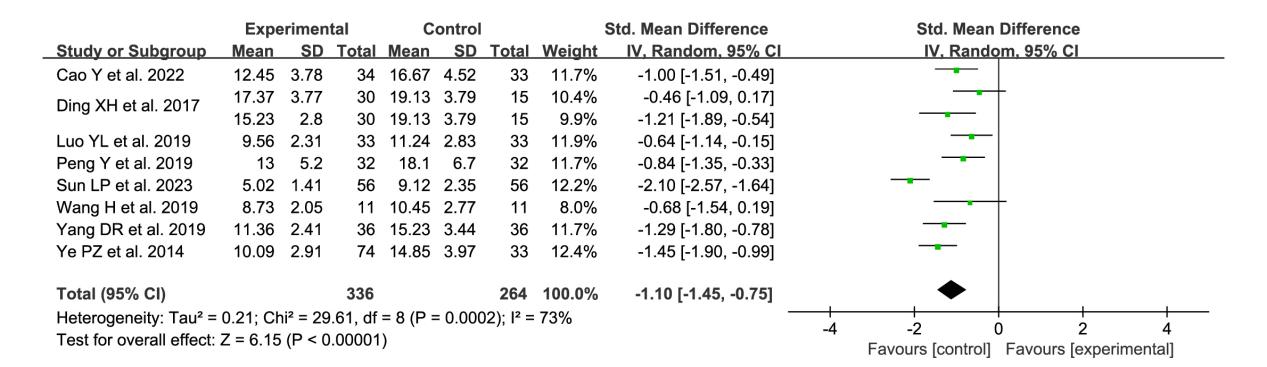


Fig 2.1.4. The forest plot of the time of indwelling urinary catheter(SMD)

**
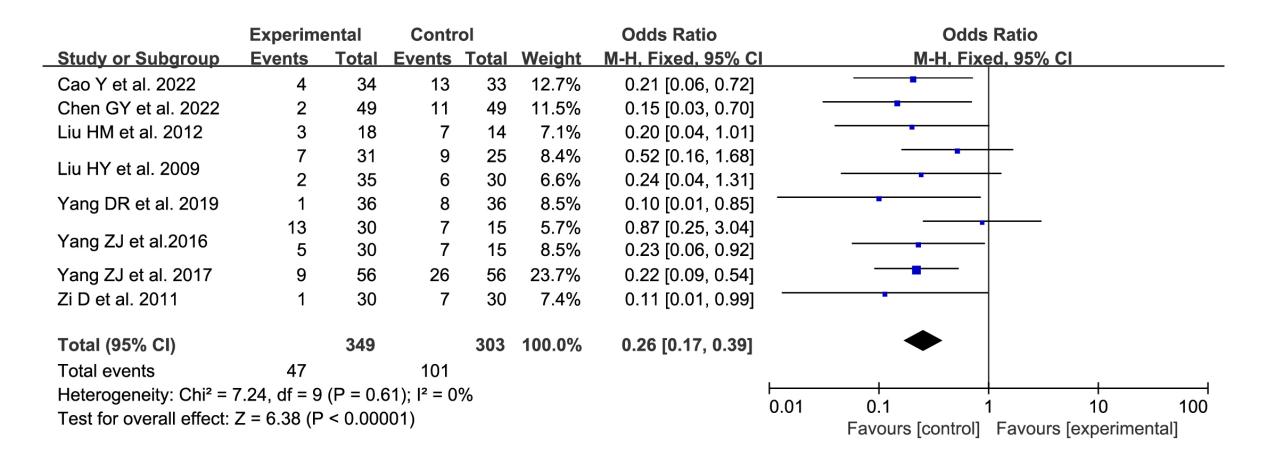
**

Fig 2.1.5. The forest plot of the incidence of UR(OR).

- 1. **The cut-and-complement method**

| Indicators | before metatrim | *P* | To trim | after metatrim | *P* |
| --- | --- | --- | --- | --- | --- |
| The total effective rate | 0.328（0.197，0.460） | *0.000* | 6 | 0.206（0.058，0.355） | *0.006* |
| The rate of urinary tract infections | -1.342（-2.581，-0.104） | *0.034* | 3 | 0.261(0.076,0.901) | *0.034* |
| The residual urine volume | -1.655（-2.063，-1.247） | *0.000* | 5 | -1.988（-2.426，-1.550） | *0.000* |
| The time of indwelling urinary catheter | -1.115（-1.467，-0.763） | *0.000* | 2 | -1.273（-1.618，-0.928） | *0.000* |
| The incidence of UR | -0.861（-1.177，-0.546） | *0.000* | 13 | 0.423（0.308，0.579） | *0.000* |

- 1. **Item-by-item exclusion study**

Fig 2.3.1. The total effective rate.

Fig 2.3.2. The urinary tract infection rates.

Fig 2.3.3. The residual urine volume.

Fig 2.3.4 The time of indwelling urinary catheter.

Fig 2.3.5. The incidence of UR.

1. **Results of subgroup analysis**
   1. **Acupucture group and electroacupuncture**
      1. **The total effective rate**

**
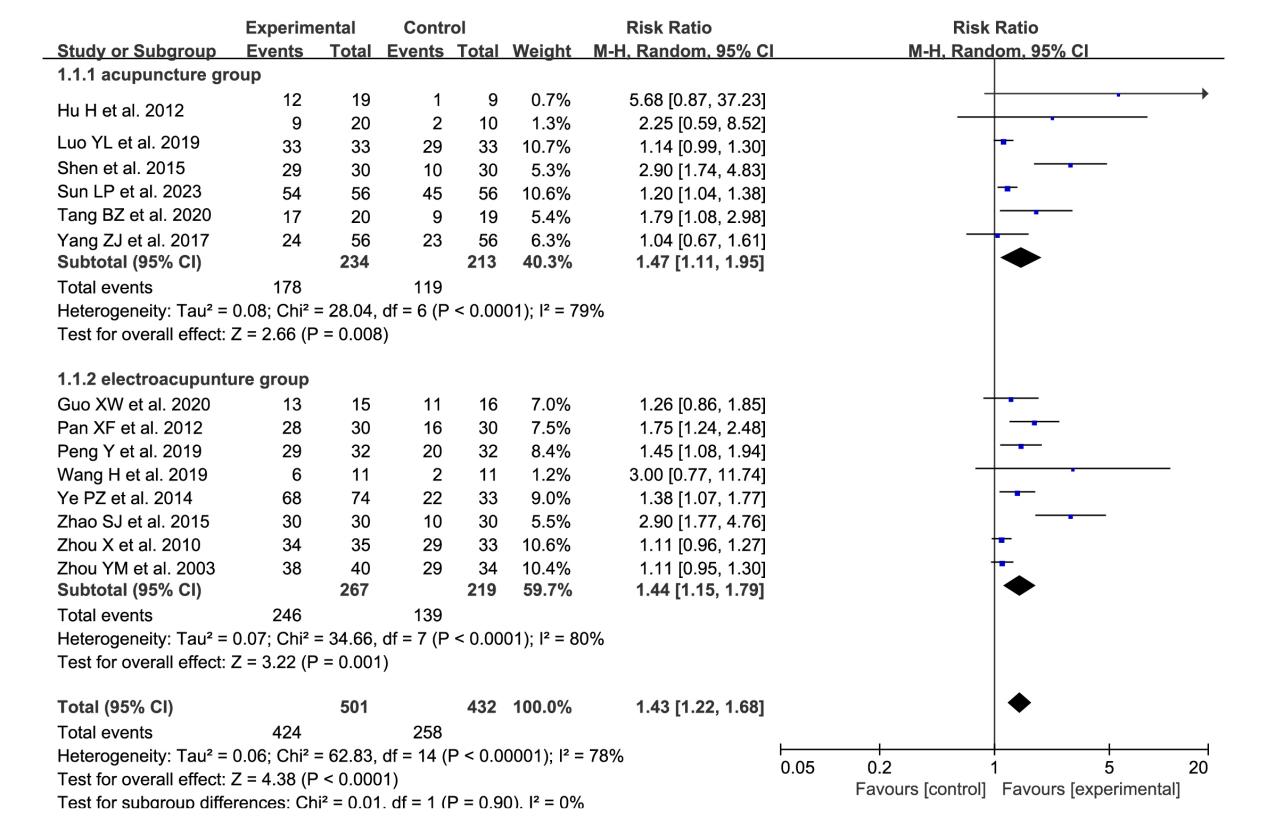
**

Fig 3.1.1. The forest plot of the total effective rate for subgroup analysis.

- - 1. **The residual urine volume**

**
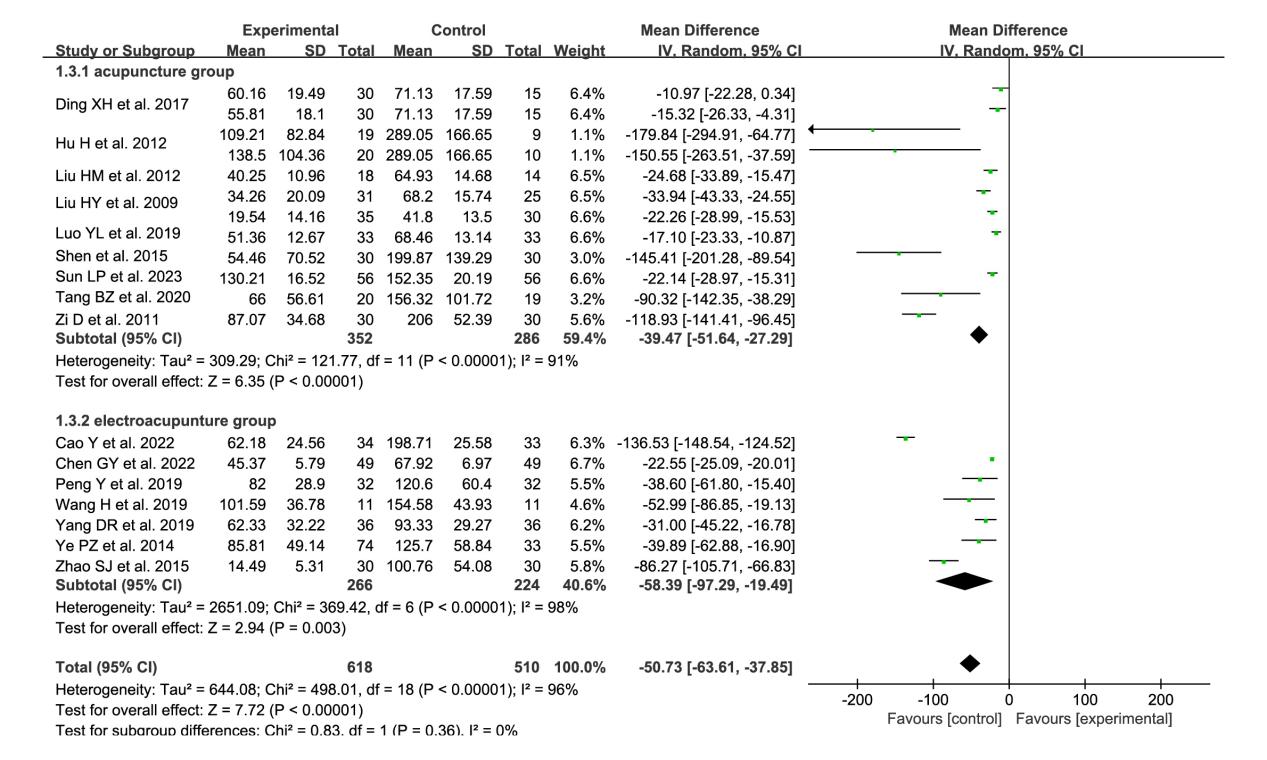
**

Fig 3.1.2. The forest plot of the residual urine volume for subgroup analysis.

- - 1. **The time of indwelling urinary catheter**

**
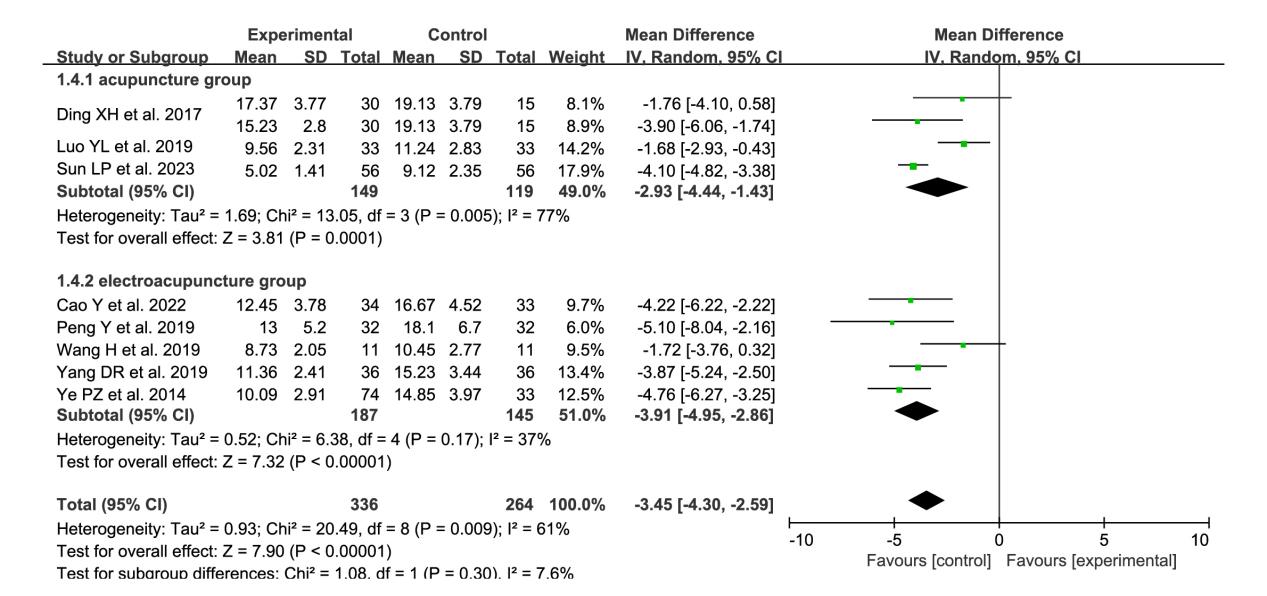
**

Fig 3.1.3. The forest plot of the time of indwelling urinary catheter for subgroup analysis.

- - 1. **The incidence of UR**


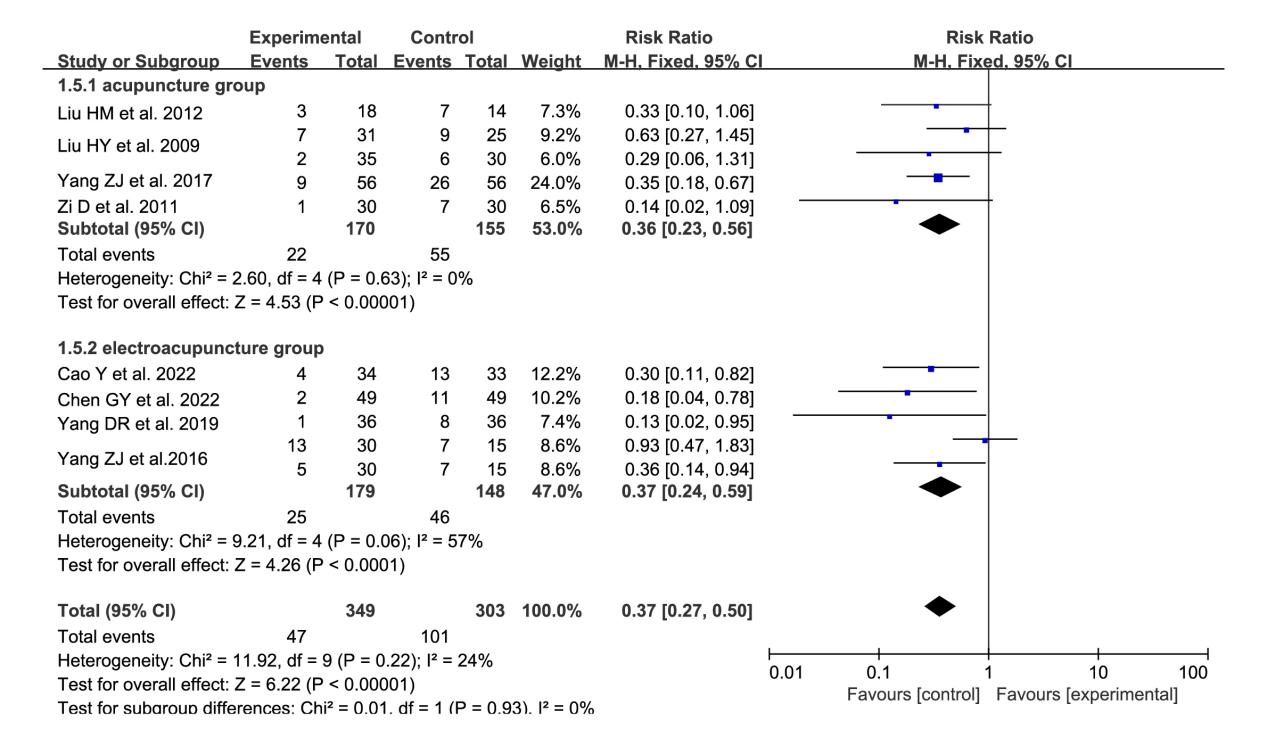


Fig 3.1.4. The forest plot of the the incidence of UR for subgroup analysis.

- 1. **Confirmed postoperative UR group and postoperative prophylactic treatment of UR group**
     1. **The total effective rate**


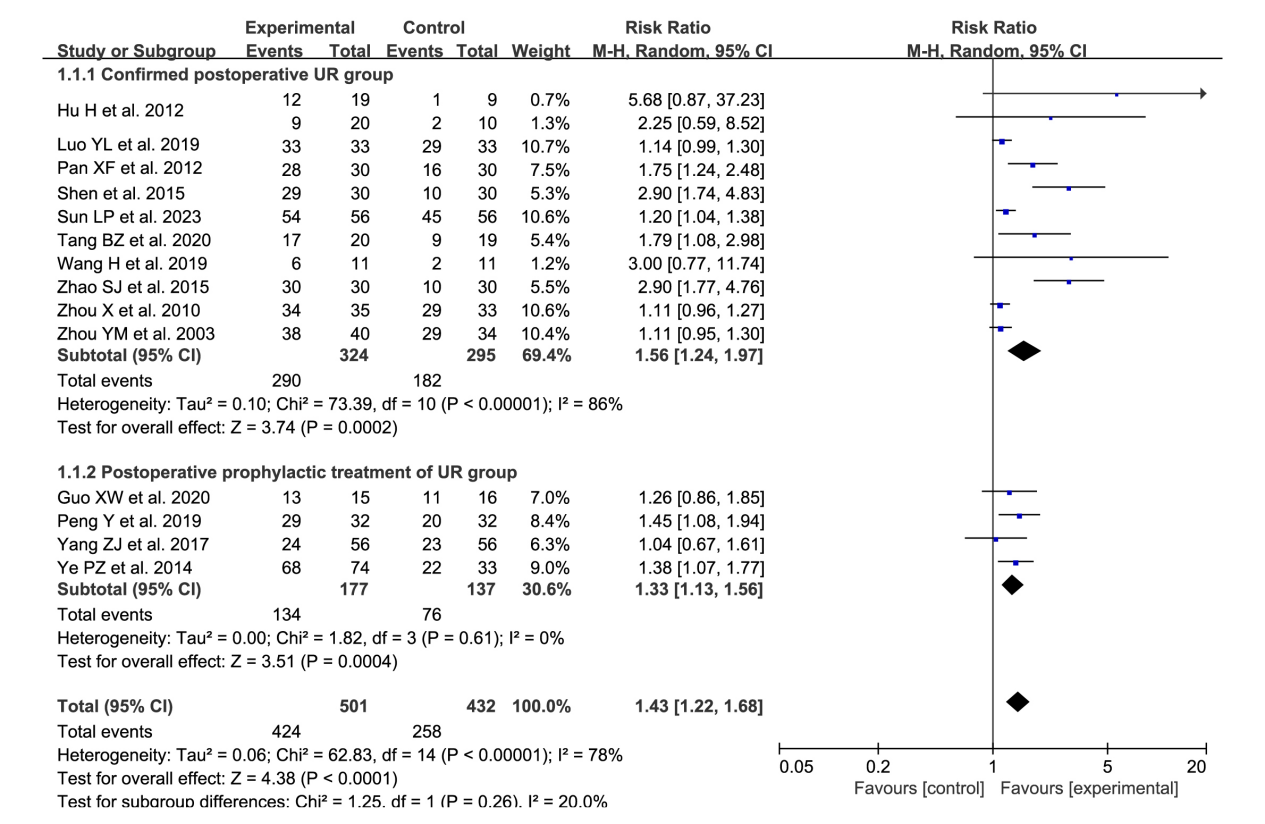


Fig 3.2.1. The forest plot of the total effective rate for subgroup analysis.

- - 1. **The residual urine volume**


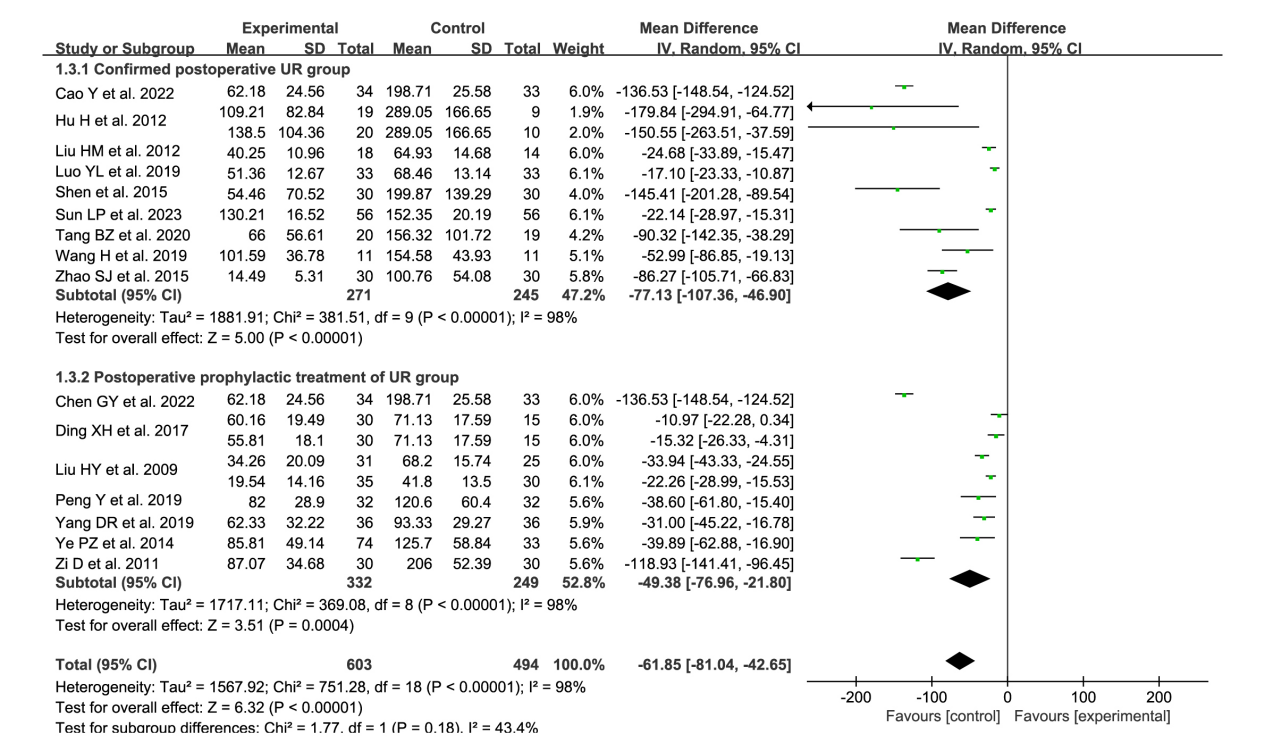


Fig 3.2.2. The forest plot of the residual urine volume for subgroup analysis.

- - 1. **The time of indwelling urinary catheter**


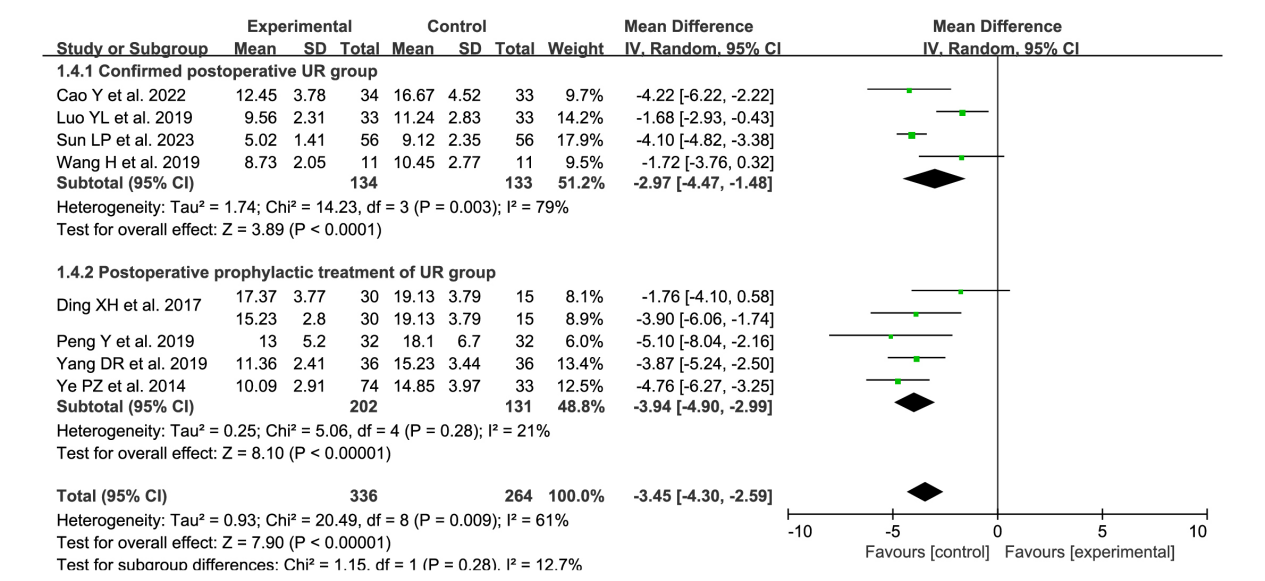


Fig 3.2.3. The forest plot of the time of indwelling urinary catheter for subgroup analysis.

- - 1. **The incidence of UR**


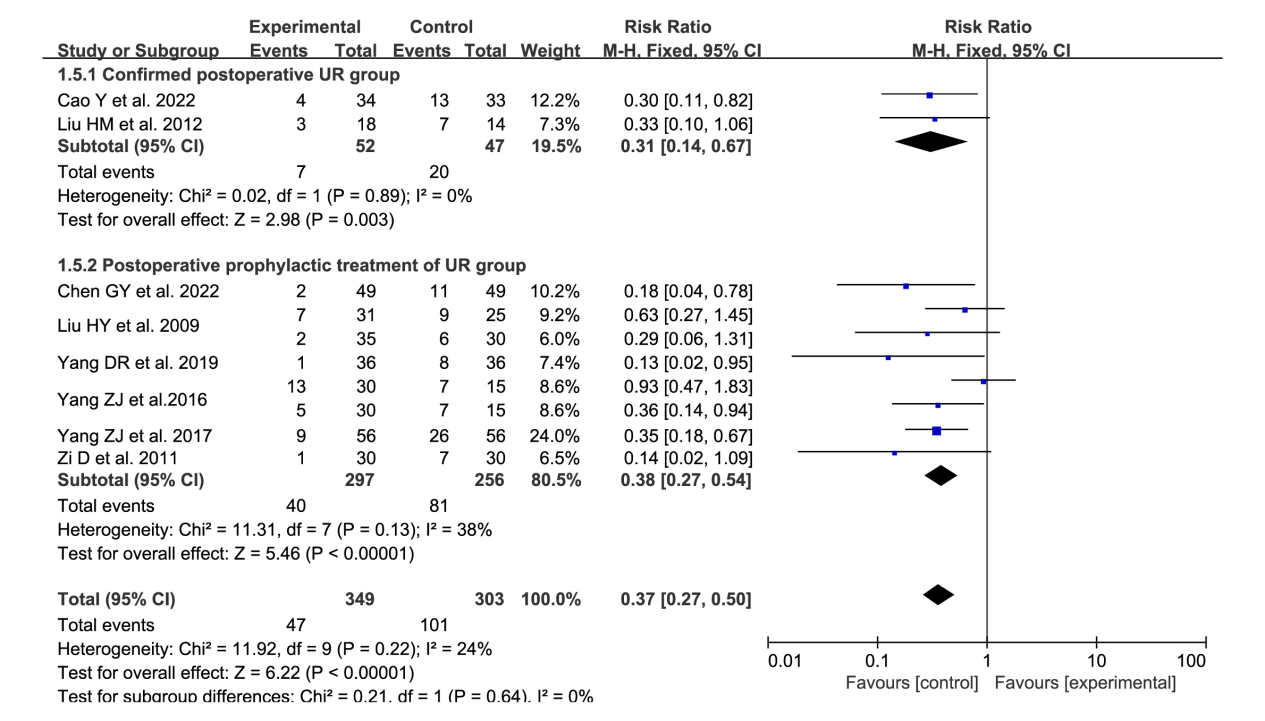


Fig 3.2.4. The forest plot of the the incidence of UR for subgroup analysis.
